# TMEM16A in Cystic Fibrosis: Activating or Inhibiting?

**DOI:** 10.3389/fphar.2019.00003

**Published:** 2019-01-29

**Authors:** Karl Kunzelmann, Jiraporn Ousingsawat, Inês Cabrita, Tereza Doušová, Andrea Bähr, Melanie Janda, Rainer Schreiber, Roberta Benedetto

**Affiliations:** ^1^Institut für Physiologie, Universität Regensburg, Regensburg, Germany; ^2^Department of Pediatrics, Second Faculty of Medicine, University Hospital Motol, Charles University in Prague, Prague, Czechia; ^3^Institute of Molecular Animal Breeding and Biotechnology, Ludwig-Maximilians-Universität München, Munich, Germany; ^4^Innere Medizin I, Klinikum Rechts der Isar der TU München, München, Germany

**Keywords:** TMEM16A, anoctamin 1, mucus secretion, cystic fibrosis, asthma, COPD, Ca^2+^ signaling

## Abstract

The inflammatory airway disease cystic fibrosis (CF) is characterized by airway obstruction due to mucus hypersecretion, airway plugging, and bronchoconstriction. The cystic fibrosis transmembrane conductance regulator (CFTR) chloride channel is dysfunctional in CF, leading to defects in epithelial transport. Although CF pathogenesis is still disputed, activation of alternative Cl^−^ channels is assumed to improve lung function in CF. Two suitable non-CFTR Cl^−^ channels are present in the airway epithelium, the Ca^2+^ activated channel TMEM16A and SLC26A9. Activation of these channels is thought to be feasible to improve hydration of the airway mucus and to increase mucociliary clearance. Interestingly, both channels are upregulated during inflammatory lung disease. They are assumed to support fluid secretion, necessary to hydrate excess mucus and to maintain mucus clearance. During inflammation, however, TMEM16A is upregulated particularly in mucus producing cells, with only little expression in ciliated cells. Recently it was shown that knockout of TMEM16A in ciliated cells strongly compromises Cl^−^ conductance and attenuated mucus secretion, but does not lead to a CF-like lung disease and airway plugging. Along this line, activation of TMEM16A by denufosol, a stable purinergic ligand, failed to demonstrate any benefit to CF patients in earlier studies. It rather induced adverse effects such as cough. A number of studies suggest that TMEM16A is essential for mucus secretion and possibly also for mucus production. Evidence is now provided for a crucial role of TMEM16A in fusion of mucus-filled granules with the apical plasma membrane and cellular exocytosis. This is probably due to local Ca^2+^ signals facilitated by TMEM16A. Taken together, TMEM16A supports fluid secretion by ciliated airway epithelial cells, but also maintains excessive mucus secretion during inflammatory airway disease. Because TMEM16A also supports airway smooth muscle contraction, inhibition rather than activation of TMEM16A might be the appropriate treatment for CF lung disease, asthma and COPD. As a number of FDA-approved and well-tolerated drugs have been shown to inhibit TMEM16A, evaluation in clinical trials appears timely.

## Introduction

The inflammatory airway disease cystic fibrosis (CF) is characterized by airway obstruction due to mucus hypersecretion, mucus plugging, and bronchoconstriction. The cystic fibrosis transmembrane conductance regulator (CFTR) chloride channel is dysfunctional in CF, leading to a loss of fluid secretion and probably impaired bicarbonate transport, along with Na^+^ hyperabsorption (Boucher, 2007; Stoltz et al., 2015). Dehydration of the airway surface (periciliary) fluid layer (ASL) covering the airway epithelium is thought to be the crucial factor leading to abnormal rheological properties of the mucus and occlusion of smaller airways (Boucher, 2007). Independent of the underlying precise molecular defect, tremendous effort was put into identification of small molecules, and natural compounds that would correct the basic defect by restoring CFTR function (Amaral and Kunzelmann, 2007; Verkman and Galietta, 2009). This, however, turned out to be a long and stony path that has finally provided effective drugs that correct and potentiate mutant CFTR. Moreover, the search is on to identify compounds that inhibit salt hyperabsorption in CF, and to find molecules that activate alternative Cl^−^ secretory pathways. What has not been in the minds of CF researchers is to look for inhibitors of certain types of Cl^−^ channels as a treatment for inflammatory airway disease (Li et al., 2017).

## Still Unresolved: The Pathogenesis of CF

Lack of appropriate Cl^−^ secretion due to defective CFTR was long regarded as the essential, if not only cause for CF lung disease. However, a pathogenic concept proposing Na^+^ hyperabsorption relative to attenuated Cl^−^ secretion, leading to airway dehydration and mucus plugging, has also been put forward in a number of studies (Boucher et al., 1986; Grubb et al., 1994; Mall et al., 1998a, 2004). We detected enhanced Na^+^ conductances in nasal *ex vivo* tissue and freshly isolated intestinal cells from CF patients (Mall et al., 1998a, 2000). Along this line, reduced ASL would lead to thickened airway mucus, airway plugging and impaired mucociliary clearance with subsequent chronic bacterial infections. Yet, this concept has been questioned by Welsh and collaborators as well as other investigators, who did not find evidence for Na^+^ hyperabsorption. In contrast, reduced airway Na^+^ absorption in CF was claimed, leading to salt accumulation in the ASL, which under normal conditions might be even hypotonic when compared with the interstitial fluid. Thus, hypertonic ASL was blamed to inactivate ß-defensins, thereby causing a predisposition toward bacterial infections (Zabner et al., 1998; Chen et al., 2010; Itani et al., 2011). In contrast, the Boucher team and others found neither evidence for a hypotonic ASL under normal conditions, nor any salt concentration (hypertonic ASL) in CF airways (Matsui et al., 1998). Given the fact that the airway epithelium is relatively leaky and has a large hydraulic conductivity, it appears somewhat unlikely that it maintains a large transepithelial osmotic gradient.

A similar controversy arose around the pH value of the ASL. It had been shown that CFTR is permeable for bicarbonate (${\text{HCO}}_{3}^{-}$), or contributes to ${\text{HCO}}_{3}^{-}$ transport as a Cl^−^ recycling pathway in a number of epithelial organs [reviewed in (Kunzelmann et al., 2017)]. To what extend ${\text{HCO}}_{3}^{-}$ is conducted by CFTR or rather operates indirectly as a Cl^−^ recycling channel that drives ${\text{HCO}}_{3}^{-}$ secretion by Cl^−^/${\text{HCO}}_{3}^{-}$ exchangers, is still a matter of debate. At any rate, Smith and Welsh were among the first to show defective cAMP-induced bicarbonate secretion in airways of CF patients (Smith and Welsh, 1992), while others showed that CFTR is permeable for ${\text{HCO}}_{3}^{-}$ (Poulsen et al., 1994; Tang et al., 2009). It should be noted that patch clamp and other types of experiments with isotonic concentrations of ${\text{HCO}}_{3}^{-}$ are not trivial and may be compromised by pH fluctuations (Kunzelmann et al., 1991). Attenuated fluid/${\text{HCO}}_{3}^{-}$ secretion in CF airways was shown to have adverse effects on the biophysical properties of airway mucus (Trout et al., 1998). Quinton and others provided further evidence that bicarbonate transport is essential for proper mucus release and viscosity (Choi et al., 2001; Quinton, 2001). In fact, ${\text{HCO}}_{3}^{-}$ transport is impaired in a number of different epithelial tissues derived from CF patients (Kunzelmann et al., 2017). Importantly, human lung pathology was brilliantly reproduced in a CF pig model. Using this CF pig model, reduced airway surface liquid pH, impaired bacterial killing, and mucus abnormalities were demonstrated (Pedersen et al., 1999; Stoltz et al., 2010; Pezzulo et al., 2012; Hoegger et al., 2014). Interestingly, Hoegger et al. demonstrated abnormal mucociliary transport in CF in submerged epithelia, which somewhat questions the role of surface dehydration in CF (Hoegger et al., 2014). In sharp contrast to these results, Schultz and coworkers found no evidence for acidic airway surface liquid pH in lungs of CF children, using a novel optical pH probe and a specialized bronchoscope (Schultz et al., 2017).

Stick and Schulz claimed that only a small fraction of infants diagnosed early with CF through the Australian AREST CF early surveillance program, present lower airway infection (Stick and Schultz, 2018). This may support the “inflammation first” concept, proposing that inflammation in CF lungs is present early in life and clearly before airway infection (Khan et al., 1995; Doring and Worlitzsch, 2000). Thus, CF epithelia appear to be in an inflammatory, pro-proliferative, and constantly remodeling state, even without any bacterial infection (Hajj et al., 2007; Rottner et al., 2007, 2009; Martins et al., 2011). CF epithelial cells were also shown to have a compromised anti-oxidant defense by superoxide dismutase (Rottner et al., 2011). Notably, some CFTR knockout mouse models do show signs of lung/airway inflammation even in the absence of mucus obstruction (Tirkos et al., 2006; Wilke et al., 2011). Moreover, airways of newborn CF pigs demonstrate developmental defects, such as cartilage abnormalities, muscle bundles, and smaller airways, which may support progression into a CF lung disease (Chen et al., 2010; Stoltz et al., 2010; Klymiuk et al., 2012). Considerable insight into epithelial ion transport and its defect in CF, was also obtained in studies with wt and transgenic mice, although mouse airways show differences in structure, distribution of submucosal glands and contribution of CFTR to Cl^−^ transport. While identical ion channels and transporters are expressed in human and mouse airway, intestinal epithelium, and other epithelial organs, the course of lung disease is mild in CF mice and airway plugging is not observed (Wilke et al., 2011). Nevertheless, because of the available broad range of tools in mouse genetics, valuable insights into organ physiology and pathological changes in CF have been gained in the different mouse CF models.

## Is Abnormal Ion Transport Relevant in CF or Only an Epiphenomenon?

Others argue that airway inflammation is second to the transport defect and to bacterial colonization (Ribeiro et al., 2005). At any rate, the most impressive clinical symptom in CF lung disease is accumulation of large amounts of airway mucus. Yet, are low pH, Na^+^ hyperabsorption, or lack of Cl^−^ secretion are truly the cause for mucus plugging and obstruction of airways? If we compare the changes in absorptive and secretory ion transport present in ßENaC-overexpressing mice (Mall et al., 2004), with the changes in ion transport in kcne3 knockout mice (Preston et al., 2010), or mice lacking TMEM16A in ciliated epithelial cells (Benedetto et al., 2017), the contribution of ion transport appears less clear. In the three mouse models we find a shift toward enhanced net absorptive transport: (i) pronounced increase in Na^+^ absorption with unchanged Cl^−^ secretion (Mall et al., 2004), (ii) milder increase in Na^+^ absorption but strongly reduced Cl^−^ secretion (Preston et al., 2010), (iii) partial reduction in Na^+^ absorption but pronounced inhibition of Cl^−^ secretion (Benedetto et al., 2017) (Table 1). Only the ßENaC mice develop a CF like lung phenotype, which may suggest that indeed Na^+^ absorption by ENaC is rate limiting and determines the direction of net flux of ions and water through an otherwise relatively leaky airway epithelium (Cotton et al., 1983; Donaldson and Boucher, 2007). This may explains why loss of Cl^−^ secretion in the airways of adult *kcne3* and *tmem16A* knockout mice does not lead to a CF like lung phenotype.

**Table 1:** Ion transport assessed by the measurement of short circuit currents in different adult mouse models [wild type (wt) littermates vs. transgenic (trans) mice].

**Mouse model**	**Amiloride-sensitive I**_****sc****_ **(μA/cm**^****2****^**)**			**cAMP-activated I**_****sc****_			**Ca**^****2+****^**-activated I**_****sc****_			**Phenotype**
	**wt**	**trans**	**%↓↑**	**wt**	**trans**	**%↓↑**	**wt**	**trans**	**%↓↑**	
ßENaC overexpressing mouse (Mall et al., 2004)	22	59	↑168	25	27	↑11	79	84	↑6	CF-like lung disease
*Kcne3*-knockout mouse (Preston et al., 2010)	48	84	↑ 75	83	17	↓80	294	50	↓83	No lung phenotype
FOXJ1-Cre-TMEM16Aflox/flox mice (Benedetto et al., 2017)	104	49	↓ 47	99	58	↓42	176	74	↓58	No lung phenotype

*Shown are approximate I_sc_ values. %↓↑, percent decrease or increase in unidirectional ion transport*.

**Figure 1:**
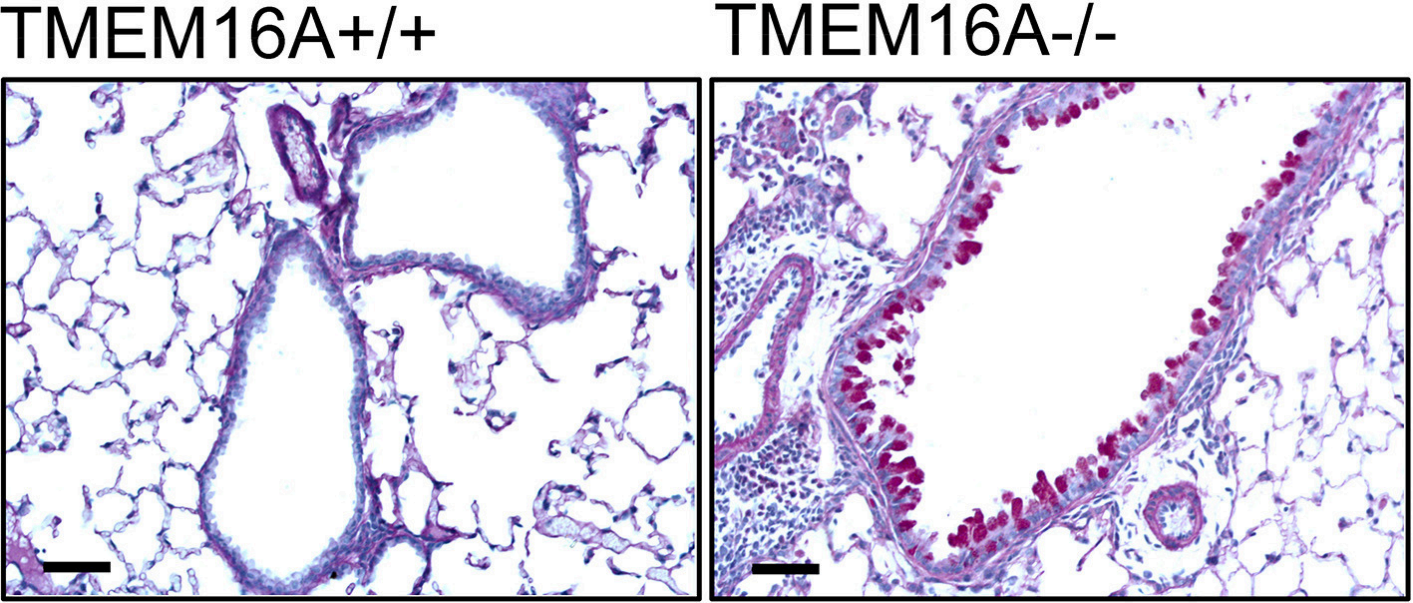
Accumulation of mucus in airways of TMEM16A^−/−^ mice. Mucus staining (PAS) in bronchi of wild type littermates (TMEM16Aflox/flox; TMEM16A^+/+^) and mice with an airway epithelial-specific knockout of TMEM16A (FOXJ1-Cre-TMEM16Aflox/flox; TMEM16A^−/−^). Strong accumulation of mucus in airway epithelial cells is observed in TMEM16A^−/−^ mice. Bars indicate 20 μm (Benedetto et al., 2019).

As discussed below, basal airway mucus secretion is attenuated in mice lacking TMEM16A in ciliated epithelial cells. This leads to mucus accumulation in secretory epithelial cells (Benedetto et al., 2019) (Figure 1). The mechanism of crosstalk between the two cell types is currently unclear. However, it is likely that ciliated cells secrete a factor that is required to release mucus from club cells. This factor could be ATP, which is found in the airway surface liquid, and which is assumed to control basal mucus secretion. Evidence has been presented that TMEM16A supports vesicular/granular exocytosis and subsequent insertion of transmembrane proteins into the plasma membrane. Moreover, it was also shown to control paracrine release of inflammatory mediators (Benedetto et al., 2016, 2017, 2019; Cabrita et al., 2017). We would favor a pathogenic mechanism that initiates the disease by intrinsic inflammation caused by dyslocalization/dysfunction of CFTR, and in the absence of bacterial infection. Intrinsic inflammation is followed by upregulation of TMEM16A, particularly in mucus producing cells, with consecutive mucus hyperproduction/hypersecretion. Accordingly, inflammation and mucus hyperproduction/hypersecretion should be pharmacologically targeted. Along this line it appears noteworthy that treatment with the anti-inflammatory drug ibuprofen was able to rescue trafficking mutant F508del-CFTR (Carlile et al., 2015).

## Correcting and Potentiating CFTR

Pharmacological restoration of defective CFTR chloride transport has been the primary goal over the past decade and has led to considerable success in correcting the gating mutant G551D-CFTR by the potentiator compound VX-770 (ivacaftor, kalydeco) (Van Goor et al., 2009; Accurso et al., 2010). G551D accounts for only a small fraction (about 5%) of all CF cases (De Boeck et al., 2014). Thus, US-based Vertex developed the small molecule CFTR-corrector VX-809 (lumacaftor), a compound that is able to rescue a fraction of the class two mutant F508del-CFTR *in vitro* (Van Goor et al., 2011; Pranke et al., 2017). Large phase 3 studies demonstrated a moderate improvement in the percentage of predicted FEV1 between 2.6 and 4.0% (Wainwright et al., 2015). Studies with next generation compounds, e.g., VX-661 (Tezacaftor), reported improvement in FEV1 in the range of 7% (Rowe et al., 2017; Taylor-Cousar et al., 2017; Donaldson et al., 2018). Recent clinical trials reported great therapeutic success with triple combinations of the corrector VX661 with VX-445 or VX-659, together with the potentiator VX-770. The combinations increased the percentage of predicted FEV1 by more than 10% (Davies et al., 2018; Keating et al., 2018). Despite such great success in correcting mutant CFTR, critical voices were raised regarding a combinatorial drug treatment. For example, adverse effects of VX-770 were reported on VX-809-corrected F508del-CFTR in the combinatorial preparation Orkambi. Moreover, long-term effects of these drugs on CFTR expression could be negative (Cholon et al., 2014; Veit et al., 2014; Chin et al., 2018). Finally, the costs for treatment by these drugs are often prohibitive (Ferkol and Quinton, 2015). Thus, there is a need for alternative drug treatments.

A number of other strategies to correct biosynthesis of misfolded CFTR are currently under investigation Recent screening efforts identified the translation initiation factor 3a (eIF3a) as a potentially druggable central hub for the biogenesis of CFTR (Hutt et al., 2018). FDA-approved histone deacetylase (HDAC) inhibitors such as panobinostat (LBH-589) and romidepsin (FK-228) can help to correct misfolded CFTR, particularly in combination with other correctors such as VX809 (Angles et al., 2018). Promising results were also obtained with combinations of pharmacological chaperones with different sites of action, such as VX-809, RDR1, and MCG1516A (Carlile et al., 2018). All these recent results are rather encouraging, however, as not every CFTR mutation is accessible to such a CFTR-based therapy, activation of other airway epithelial Cl^−^ channels was proposed to compensate for defective CFTR (De Boeck and Amaral, 2016).

## Airway Chloride Channels: SLC26A9

**Figure 2:**
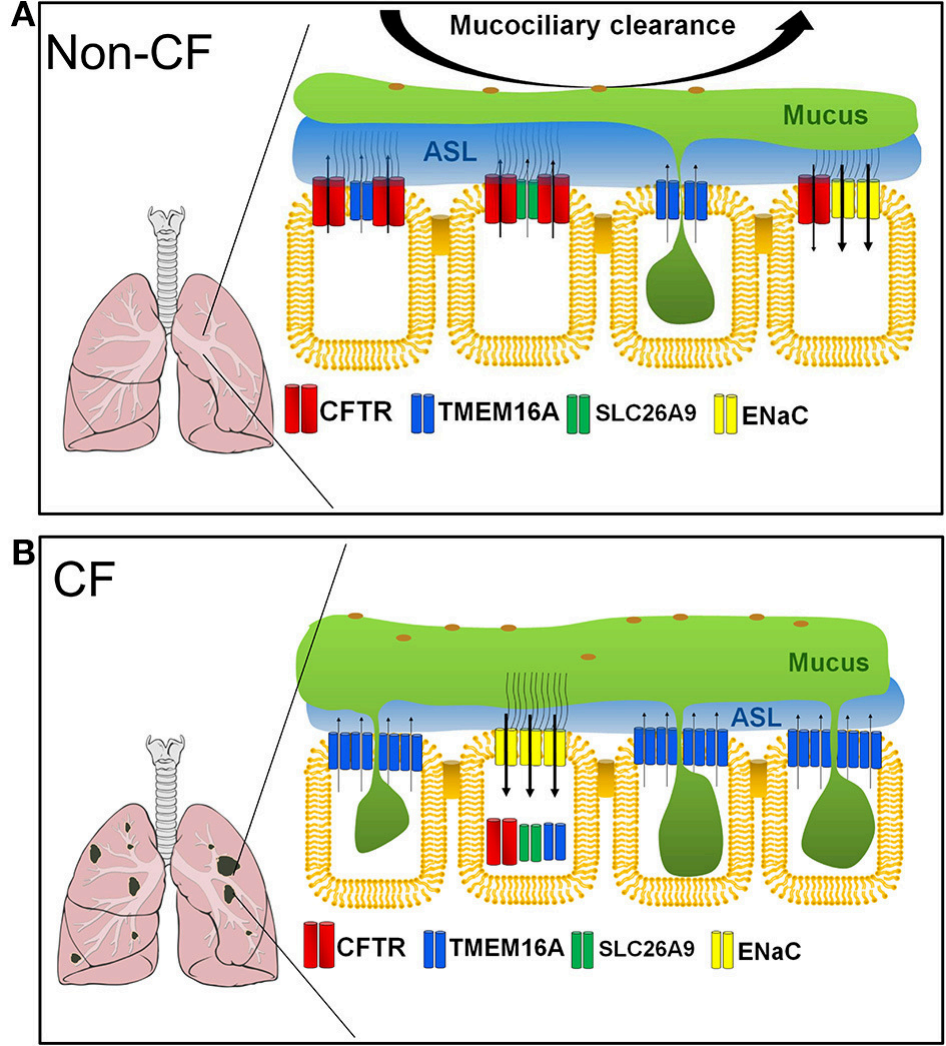
Ion channels contributing to fluid/mucus balance in non-CF and CF airways. **(A)** In non-CF airways, CFTR is expressed in ciliated epithelial cells and ionocytes (not shown). Ca^2+^ activated Cl^−^ channels are sparsely expressed in both ciliated and secretory club and goblet cells. SLC26A9, TMEM16A and CFTR located in ciliated epithelial cells and probably in ionocytes are in charge of fluid secretion, while TMEM16A expressed in secretory club/goblet cells support mucus secretion. Epithelial Na^+^ channels reabsorb Na^+^ thereby causing fluid absorption. **(B)** In inflamed CF airways, CFTR is dysfunctional and often dislocated intracellularly together with SLC26A9 and TMEM16A in ciliated epithelial cells. TMEM16A is upregulated in secretory cells strongly contributing to mucus secretion. Na^+^ absorption by ENaC is augmented. Inflammation and transport abnormalities lead to excessive mucus production/secretion, airway plugging, and reduced water secretion, strongly reducing mucociliary clearance.

**Figure 3:**
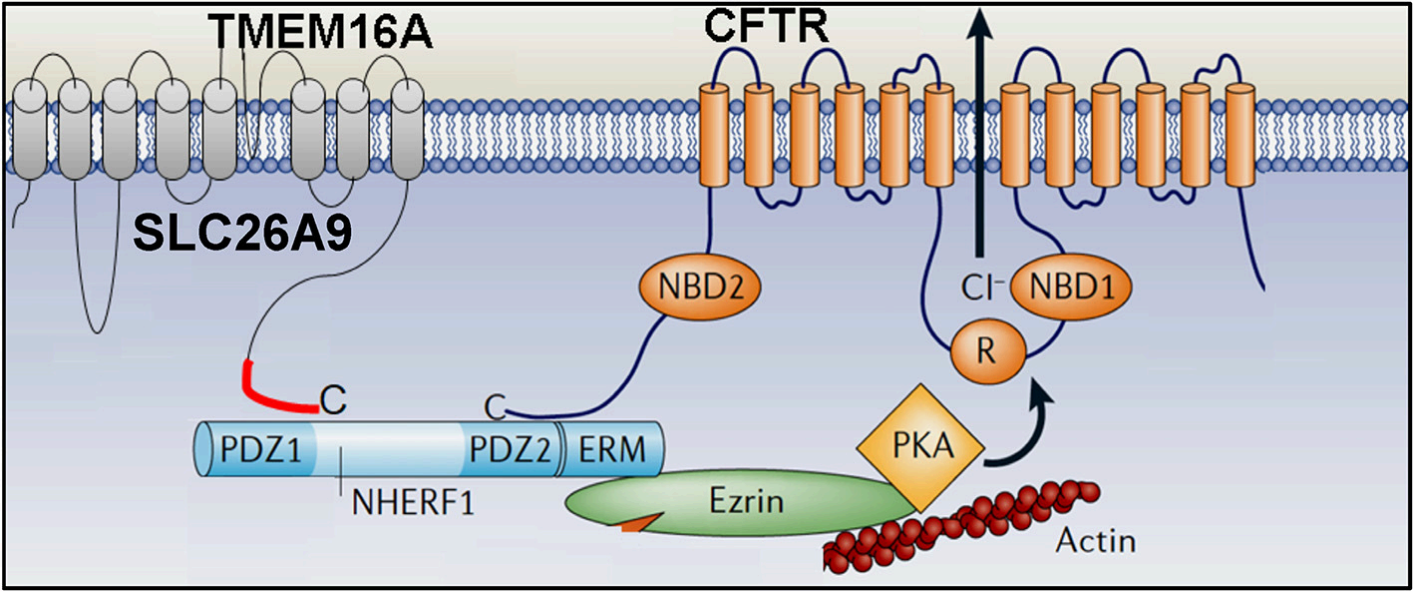
Molecular and functional relationship of CFTR, SLC26A9 and TMEM16A. Both SLC26A9 and TMEM16A interact with CFTR via their C-terminal PDZ (PSD-95/Discs-large/ZO1) -binding domains with the help of the PDZ-protein NHERF1 (Na^+^/H^+^ exchanger regulatory factor 1).

Apart from CFTR, there are two other major Cl^−^ channels present in human and mouse airways, namely SLC26A9 and TMEM16A. How much both Cl^−^ channels quantitatively contribute to production of the ASL and support mucociliary clearance is currently not known (Figure 2). SLC26 proteins typically operate as anion exchangers, but for SLC26A9 it was shown to function as a Cl^−^ channel (Mount and Romero, 2004; Ousingsawat et al., 2011b; Bertrand et al., 2017). In contrast to CFTR, SLC26A9 is spontaneously active, once inserted into the apical membrane of airway epithelial cells. However, it is also regulated by Cl^−^ feedback/WNK kinases and surprisingly is controlled by the Cl^−^ channel CFTR (Dorwart et al., 2007; Bertrand et al., 2009). SLC26A9 is likely to provide the basal Cl^−^ conductance that is found in airways in the absence of any secretagogue (Bertrand et al., 2017). Absence of basal Cl^−^ secretion in airways of CF patients carrying the type II mutation F508del-CFTR (De Boeck and Amaral, 2016), is probably due to the lack of expression of SLC26A9 in the apical membrane (Figure 2B) (El Khouri and Toure, 2014; Bertrand et al., 2017). SLC26A9 and CFTR form a complex with the help of PDZ scaffold proteins such as NHERF1 (Figure 3). When coexpressed with wtCFTR, SLC26A9 is co-trafficked together with CFTR from the ER to the plasma membrane. However, if coexpressed with F508del-CFTR, SLC26A9 will remain intracellularly and will be degraded (Bertrand et al., 2017). As shown recently, also CFTR and TMEM16A do interact via PDZ-domain proteins, and TMEM16A has an impact on plasma membrane expression of CFTR (Benedetto et al., 2017).

Furthermore, SLC26A9 may be regulated by the R domain of CFTR through STAS domain interaction (Chang et al., 2009). Indeed, SLC26A9 has been demonstrated to be a genetic modifier in CF (Sun et al., 2012; Miller et al., 2015), and the CFTR corrector VX-809 partially rescued SLC26A9, probably by facilitating trafficking of F508del-CFTR to the plasma membrane (Strug et al., 2016). Similar to TMEM16A, SLC26A9 is upregulated during airway inflammation and exposure of the airway cells to IL-13 (Anagnostopoulou et al., 2012). It will be interesting to learn more about the regulation of SLC26A9 expression, and whether SLC26A9 is upregulated in CF airways in ciliated or in mucus secreting cells. Taken together, SLC26A9 could potentially serve as an alternative Cl^−^ channel in CF, but compromised biosynthesis of CFTR carrying type two mutations need to be corrected. Identifying small molecules that would interfere with the formation of the F508del-CFTR/SLC26A9 complex could be an interesting therapeutic option in CF.

## Airway Chloride Channels: TMEM16A

TMEM16A is a Ca^2+^ activated Cl^−^ channel (CaCC) that belongs to a larger family of 10 paralogous proteins (TMEM16A-K), also called anoctamins (ANO1-ANO10) (Kunzelmann et al., 2011; Pedemonte and Galietta, 2014). The majority of these double-barreled channels are operating as phospholipid scramblases, i.e., they transport phospholipids from one site of the bilayer membrane to the other, once activated by a strong increase in intracellular Ca^2+^ (Suzuki et al., 2010). TMEM16A and B are solely CaCCs, whose structure and gating has been largely uncovered in cryo-EM studies (Paulino et al., 2017a,b). TMEM16A is typically localized in the apical plasma membrane of epithelial cells. However, it is also found to be expressed basolateral and in intracellular compartments (Schreiber et al., 2010, 2014). TMEM16F is a phospholipid scramblase that also conducts Cl^−^ and other ions (Yang et al., 2012; Grubb et al., 2013; Shimizu et al., 2013; Kunzelmann et al., 2014; Ousingsawat et al., 2015; Scudieri et al., 2015; Drumm et al., 2017; Schreiber et al., 2018). Although endogenous TMEM16 proteins are mostly localized intracellularly, overexpression of these proteins together with purinergic receptors allows partial trafficking to the plasma membrane (Tian et al., 2012a). TMEM16A is clearly the epithelial airway CaCC (Benedetto et al., 2017), but a number of other TMEM16 paralogues are also coexpressed in mouse airways and in human large and small bronchi, bronchiole and alveoli, such as TMEM16C, F, J (Kunzelmann et al., 2012). Particularly TMEM16F may participate as well in epithelial Cl^−^ transport.

## Upregulation of TMEM16A During Inflammatory Lung Disease: Good or Bad?

TMEM16A is strongly upregulated during inflammation, a fact that was utilized to identify the molecular nature of CaCC (Galietta et al., 2002; Caputo et al., 2008). TMEM16A is strongly upregulated in CF and asthma, which parallels goblet cell metaplasia and mucus hypersecretion (Huang et al., 2012; Kondo et al., 2017), and is also upregulated by bacterial components (Caci et al., 2015). Upregulation of TMEM16A is predominant in mucus producing cells and to a much lesser degree in ciliated epithelial cells (Huang et al., 2012; Scudieri et al., 2012). Expression of TMEM16A is almost undetectable by immunocytochemistry in normal adult human and mouse airways; although CaCC is clearly present (Huang et al., 2009, 2012; Benedetto et al., 2017). While this may be explained by the limited sensitivity of the available antibodies, it also raises questions as to what degree other members of the TMEM16 family might participate in CaCC. As mentioned above, TMEM16C, F, and J are also expressed in mouse airway epithelium (Kunzelmann et al., 2012). On the other hand, knockout of TMEM16A completely abolished CaCC activated by purinergic stimulation (Benedetto et al., 2017). We will discuss below that TMEM16A has a strong impact on intracellular Ca^2+^ signals triggered by stimulation of G-protein coupled receptors (GPCRs), which then activate phospholipase C, increase inositol trisphosphate and intracellular Ca^2+^ (Kunzelmann et al., 2016).

**Figure 4:**
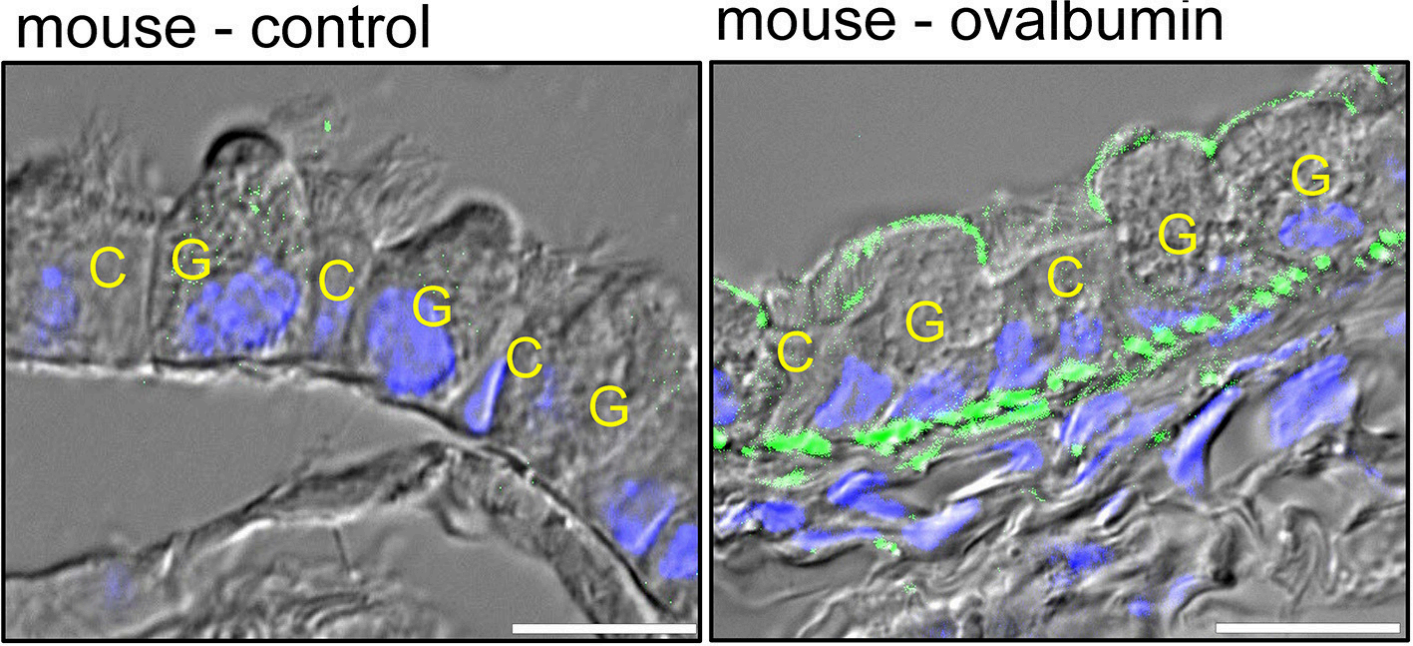
Predominant expression of TMEM16A in mucus producing cells. Distal airways of a control mouse and an ovalbumin-sensitized (asthmatic) mouse. Expression of TMEM16A (green fluorescence) is hardly detectable in control airways, but is clearly detectable in mucus producing club/goblet cells (G) of asthmatic mice with inflamed airways. Little expression of TMEM16A is found in ciliated epithelial cells (C). Blue, DAPI staining of nuclei. Bars indicate 20 μm (Benedetto et al., 2019).

As mentioned above, expression of TMEM16A in normal adult airways is hardly detectable by immunohistochemistry (Ousingsawat et al., 2009; Benedetto et al., 2017) (Figure 4). However, induction of airway inflammation in an ovalbumin asthma model induced a pronounced upregulation of TMEM16A in mucus producing club/goblet cells, but induced little expression in ciliated epithelial cells (Benedetto et al., 2019) (Figure 4). Ciliated epithelial cells, and particularly the recently identified ionocytes express CFTR and are in charge of fluid secretion (Montoro et al., 2018; Plasschaert et al., 2018). Expression of TMEM16A is low in ciliated cells when compared to mucus producing club and goblet cells. The contribution of TMEM16A to overall fluid secretion by the airway epithelium might therefore be limited, while it plays a central role for basal mucus secretion (Benedetto et al., 2019). This will be further outlined below.

## F508del-CFTR Attenuates Expression of TMEM16A in the Apical Membrane

A functional coupling between TMEM16A and CFTR has been described in a number of previous publications (Kunzelmann et al., 1997; Wei et al., 1999; Ousingsawat et al., 2011a). Subsequent studies reported attenuated expression of TMEM16A in the apical membrane of airway epithelial cells by coexpressed F508del-CFTR (Ruffin et al., 2013; Benedetto et al., 2017). We demonstrated that TMEM16A and CFTR directly interact through PSD-95/Dlg/ZO-1 (PDZ) domain proteins, similar to SLC26A9 (Figure 3). The functional interaction between TMEM16A and CFTR is also demonstrated by a crosstalk of intracellular Ca^2+^ and cAMP-dependent signaling. This compartmentalized crosstalk is facilitated by exchange protein directly activated by cAMP (EPAC1) and Ca^2+^ -sensitive adenylate cyclase type 1 (ADCY1). Assembly of such a local signalosome was shown to depend on the number of phospholipase C coupled GPCRs (Lerias et al., 2018).

These studies suggest a significant overlap of cAMP- and Ca^2+^ activated Cl^−^ currents. Along this line, our early studies also showed that epithelial cAMP-dependent and Ca^2+^-activated Cl^−^ currents cannot be easily separated based on apparently specific ion channel inhibitors (Kunzelmann et al., 1992; Benedetto et al., 2017; Lerias et al., 2018). Moreover, in both airway and intestinal epithelium, most of the Ca^2+^ activated Cl^−^ secretion is in fact through CFTR and not through TMEM16A Cl^−^ channels (Mall et al., 1998b; Namkung et al., 2010a; Billet and Hanrahan, 2013; Benedetto et al., 2017). The actual purinergic (ATP-activated) Cl^−^ secretion via TMEM16A is very transient, as TMEM16A deactivates quickly within 1–5 min after activation by ATP when examined in *Xenopus* oocytes (Faria et al., 2009), HEK293 cells (Tian et al., 2012b), mouse trachea (Kunzelmann et al., 2002), and human airways (Mall et al., 2003). Analysis of freshly isolated human nasal epithelial cells demonstrates ATP-induced steady-state secretion only in non-CF cells, but not in CF nasal cells. Thus, the direct contribution of TMEM16A to the epithelial secretory Cl^−^ transport is small. However, the non-transient steady component of purinergic Cl^−^ secretion that is produced by CFTR is essential for fluid secretion (Mall et al., 2003; Benedetto et al., 2017). The traffic mutant F508del is by far the most common mutation in CF that also compromises biosynthesis of TMEM16A. Therefore, the pro-secretory function of TMEM16A in CF is probably limited, and inhibition of TMEM16A may not much reduce Cl^−^ secretion in CF (Ruffin et al., 2013; Benedetto et al., 2017).

## Upregulation of TMEM16A in Airway Smooth Muscles

Upon induction of asthma, we also observed an upregulation of TMEM16A in mouse airway smooth muscle (ASM) cells (Benedetto et al., 2017; Miner et al., 2019) (Figure 4). This has already been described in a number of previous studies (Huang et al., 2009, 2012; Gallos et al., 2013; Danielsson et al., 2015). Inhibitors of TMEM16A were shown to induce hyperpolarization of ASM and airway relaxation (Yim et al., 2013; Danielsson et al., 2017; Miner et al., 2019). Airway inflammation is well-known to induce hyperresponsiveness of ASM (Brightling et al., 2002; Galli et al., 2008). Inflammatory mediators binding to GPCRs activate TMEM16A channels; depolarize the membrane voltage and cause airway contraction, a process that is upregulated in asthma (Wang et al., 2018). Expression of TMEM16A is not only upregulated in allergic asthma, but also in airway epithelial cells and probably ASM of CF patients (Caci et al., 2015). Moreover, the signaling cascade comprising GPCR - TMEM16A - intracellular Ca^2+^ is further augmented by inflammatory mediators and cholinergic stimuli.

## TMEM16A Controls Ca^2+^ Signals, Membrane Exocytosis and Mucus Secretion

**Figure 5:**
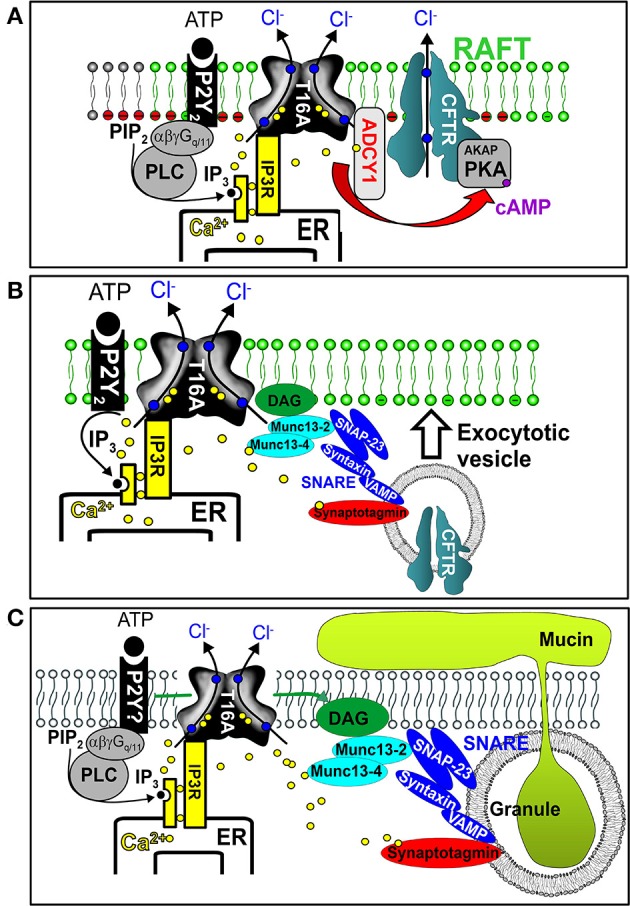
Impact of TMEM16A on intracellular Ca^2+^, exocytosis, and mucus secretion. **(A)** TMEM16A is colocalized with purinergic P2Y receptors and CFTR within the apical membrane. Stimulation of purinergic receptors leads to endoplasmic reticulum (ER) Ca^2+^ store release through IP3 receptors (IP3R). Ca^2+^ not only activates TMEM16A (T16A) but also stimulates adenylate cyclase type 1 (ADCY1) to produce cAMP and to activate CFTR via protein kinase A (PKA). TMEM16A binds to IP3 receptors and tethers the ER to the apical membrane, thereby facilitating effective compartmentalized Ca^2+^ signaling. **(B)** Effective apical Ca^2+^ signaling by TMEM16A leads to activation of the exocytic SNARE machinery insertion and improved expression of CFTR in the apical plasma membrane. **(C)** Effective apical Ca^2+^ signaling by TMEM16A leads to fusion of mucus containing granules, exocytosis, and release of mucus.

As pointed out above, membrane expression and activity of CFTR strongly relies on TMEM16A (Benedetto et al., 2017; Lerias et al., 2018). We showed that augmentation of apical Ca^2+^ signals in the presence of TMEM16A activates adenylate cyclase type 1, enhances local cAMP levels and boosts CFTR activity (Figure 5A). The enhanced plasma membrane expression of CFTR in the presence of TMEM16A may be caused by enhanced Ca^2+^ levels in the apical submembranous compartment, which triggers the exocytic machinery and membrane insertion of CFTR in ciliated epithelial cells and possibly ionocytes (Benedetto et al., 2017) (Figure 5B). A similar exocytic mechanism may apply to the process of mucus secretion by club and goblet cells. We found that ATP-induced mucus secretion by secretory cells is strongly compromised in the absence of TMEM16A. Without TMEM16A, intracellular Ca^2+^ concentrations in the apical pole of club and goblet cells are attenuated. These Ca^2+^ ions are required for basal and acute ATP-activated mucus release (Benedetto et al., 2019) (Figure 5C). ATP-dependent mucus secretion is characterized by Ca^2+^ dependent single granule docking to the apical membrane which requires Munc13 proteins and the SNARE (soluble N-ethylmaleimide–sensitive factor attachment protein receptor) machinery (Fahy and Dickey, 2010). Taken together, in healthy non-CF airways TMEM16A may support CFTR-driven fluid secretion in ciliated cells and possibly ionocytes, and supports basal mucus release by club and goblet cells. In inflammatory CF airway disease, the function of TMEM16A may be marginal in ciliated cells and ionocytes, but may be pronounced in secretory cells due to strong upregulation of expression.

**Figure 6:**
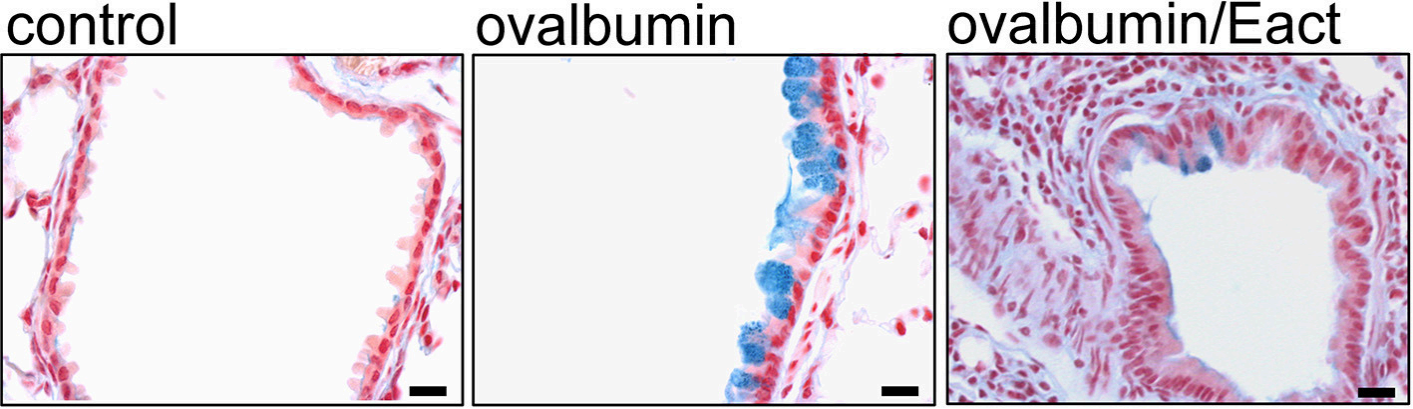
Activation of TMEM16A leads to mucus release and airway contraction. Control mouse airways show very little mucus (alcian blue). In contrast, airways from ovalbumin-sensitized mice show pronounced mucus accumulation. Acute exposure of an ovalbumin-sensitized mouse to the known activator of TMEM16A, Eact (4.8 μg, tracheal instillation), induced a rapid mucus release and airway contraction (Benedetto et al., 2019). Bars indicate 20 μm.

According to this, pharmacological activation of TMEM16A in CF and asthma patients could have adverse effects on lung function due to its prosecretory effect on mucus release. Correspondingly, we found in OVA-sensitized asthmatic mice that activation of TMEM16A by the compound Eact (Namkung et al., 2011b) induced massive mucus release and a considerable expiratory stridor, suggesting airway contraction. Airway narrowing was confirmed by analysis of the airway cross section (Benedetto et al., 2019) (Figure 6). It may be argued that Eact raises intracellular Ca^2+^ and therefore induces adverse effects independent of TMEM16A. However, increase in intracellular Ca^2+^ by Eact is expected. As outlined below, activation of TMEM16A is known to increase intracellular Ca^2+^ (Cabrita et al., 2017). Activation of TMEM16A by Eact depolarizes the membrane voltage, which leads to an increase in intracellular Ca^2+^. Notably, increase in intracellular Ca^2+^ by Eact was inhibited by 1 μM of the TMEM16A blocker niclosamide (Benedetto et al., 2019).

## Inhibitors and Activators of TMEM16A

Meanwhile a larger number of inhibitors of TMEM16A has been identified, but there is only one published group of N-aroylaminothiazole “activators” (Eact) (Namkung et al., 2011b), apart from some herbal compounds like Ginsenoside Rb1 and Resveratrol, which apparently activate TMEM16A in a Ca^2+^ independent manner (Chai et al., 2017; Guo et al., 2017) (Table 2). Enterprise therapeutics (Sussex, UK; http://www.enterprisetherapeutics.com/) is currently working on potentiators of TMEM16A, but details on potentiating molecules are not yet available. Silurian pharmaceuticals (Oakland, US; http://www.silurianpharma.com/index.php) reported brevenal to activate Ca^2+^ activated Cl^−^ channels, possibly in a Ca^2+^ independent fashion. Denufusol was developed by Inspire pharmaceuticals (later taken over by Merck). Denufosol is a deoxycytidine-uridine dinucleotide with enhanced metabolic stability, to activate purinergic P2Y2 receptors which stimulate TMEM16A (Yerxa et al., 2002) and inhibit ENaC (Kunzelmann et al., 2002) (c.f. below). An interesting group of lipids was identified originally to uncouple GPCR-mediated Ca^2+^ increase from inactivation/desensitization of Ca^2+^ activated Cl^−^ channels. These D-myo-inositol 3,4,5,6-tetrakisphosphate [Ins(3,4,5,6)P4] (Vajanaphanich et al., 1994) were synthetically modified to result in the membrane permeable analog INO4995, which was shown to inhibit ENaC (Moody et al., 2005) and to activate TMEM16A (Tian et al., 2012b). INO4995 did not increase intracellular Ca^2+^. It activated overexpressed TMEM16A directly, but potentiated ATP-dependent activation of TMEM16A expressed endogenously. Preliminary data suggested enhanced membrane localization of TMEM16A induced by INO4995 (Tian et al., 2012b) (Table 3).

**Table 2:** Inhibitors of TMEM16A.

**Inhibitor**	**IC50 (μM)**	**Structural formula**	**References**
10bm	0.03	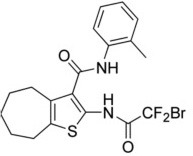	Truong et al., 2017
Monna	0.08	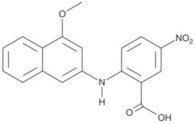	Oh et al., 2013
Niclosamide	0.7	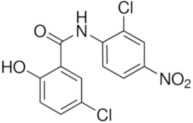	Miner et al., 2019
Ani9	0.1	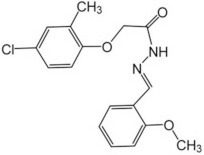	Seo et al., 2016
Tannic acid	0.323	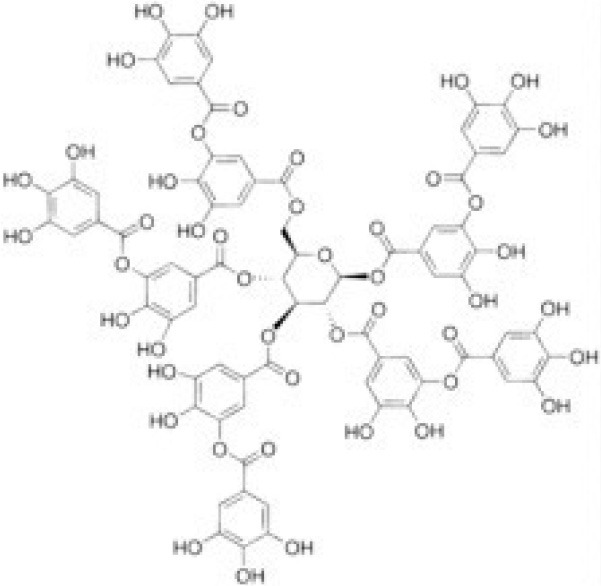	Namkung et al., 2010b
T16A-A01	1.1	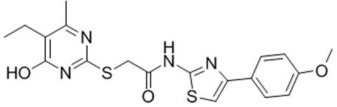	Namkung et al., 2011a; Fedigan et al., 2017
Dichlorophen	5.49	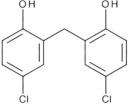	Huang et al., 2012
Idebenone	5.52	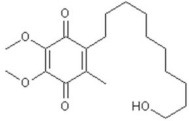	Seo et al., 2015
Shikonin	6.5	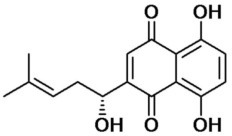	Jiang et al., 2016
Benzbromarone	9.97	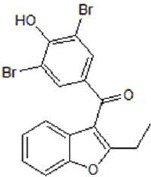	Huang et al., 2012
CaCC-A01	10	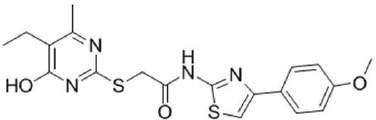	de La Fuente et al., 2007; Gianotti et al., 2016; Fedigan et al., 2017
9-Phenanthrol	12	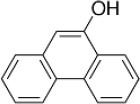	Burris et al., 2015)
Niflumic Acid	12	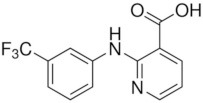	Hogg et al., 1994
Flufenamic acid	28	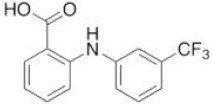	White and Aylwin, 1990
Talniflumate	–	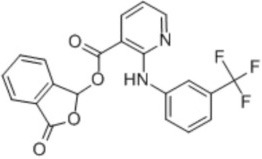	Walker et al., 2006
A9C	58	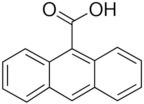	Baron et al., 1991
Dehydroandrographolide	20–30	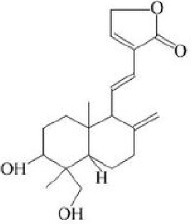	Sui et al., 2015
DIDS	10–100	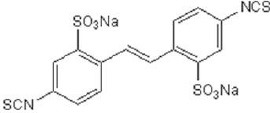	Kubitz et al., 1992
NPPB	15–150	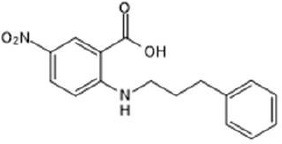	Kubitz et al., 1992; Yang et al., 2008
Rice bran extract			Sharm et al., 2017
Matrine	28 μM	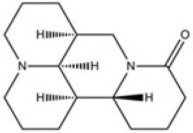	Guo et al., 2018
(Ani9 derivative) 5f	22 nM	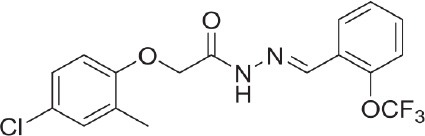	Seo et al., 2018

**Table 3:** Activators of TMEM16A.

**Activator**	**IC50 (μM)**	**Structural formula**	**References**
Brevenal	–	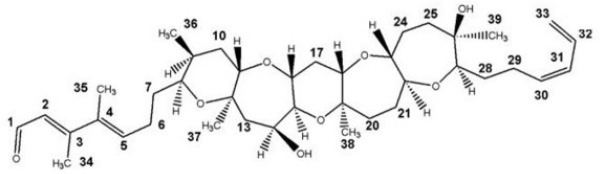	http://www.silurianpharma.com/index.php
Eact	3	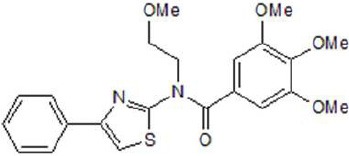	Namkung et al., 2011b
INO-4995	5	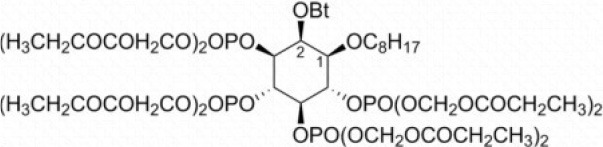	Tian et al., 2012b
Denufosol	10	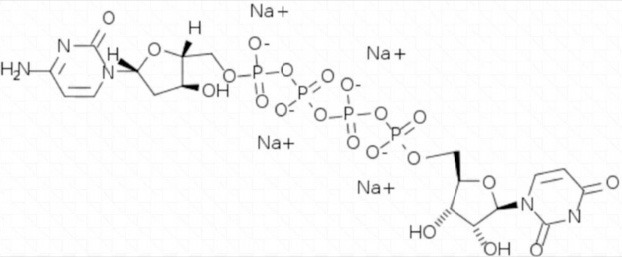	Yerxa et al., 2002
Cinnamaldehyde	10	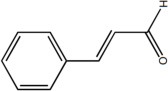	Huang et al., 2018
Ginsenoside Rb1	38.4	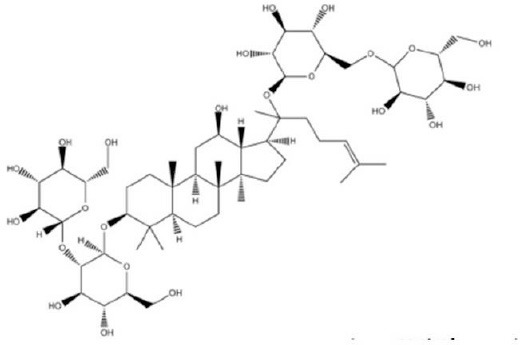	Guo et al., 2017
Resveratrol	47.9	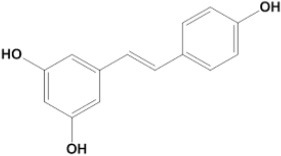	Chai et al., 2017
A9C	100–1000	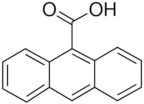	Ta et al., 2016

## Biological Effects of Inhibitors and Activators of TMEM16A

TMEM16A is broadly expressed in many epithelial and non-epithelial tissues. It is therefore expected that inhibition or activation of TMEM16A through systemic drug application might have a number of side effects. Apart from inhibiting mucus production and mucus secretion, TMEM16A-inhibitors will induce bronchorelaxation by blocking TMEM16A in airway smooth muscle (Huang et al., 2012; Gallos et al., 2013; Zhang et al., 2013; Danielsson et al., 2015; Wang et al., 2018; Miner et al., 2019). Another desired lung-specific effect of TMEM16A-inhibitors is the inhibition of release of inflammatory mediators (Knight, 2004; Danielsson et al., 2017; Benedetto et al., 2019).

Some inhibitors of TMEM16A have additional beneficial effects, such as protection from reactive oxygen species (idebenone, Villalba et al., 2010), and general inhibition of inflammation (Knight, 2004; Schreiber et al., 2018). Many studies demonstrate inhibition of proliferation and anti-cancer effects by blocking TMEM16A (Wanitchakool et al., 2014; Wang et al., 2017). General anti-hypertensive effects are likely (Namkung et al., 2010b; Heinze et al., 2014), as well as inhibition of nociception, itching, and heat perception (Cho et al., 2012; Lee et al., 2014; Pusch and Zifarelli, 2014; Deba and Bessac, 2015). Inhibition of saliva production and dry mouth may occur mediation with TMEM16A-inhibitors (Ousingsawat et al., 2009; Catalan et al., 2015). Inhibition of TMEM16A may also attenuate intestinal contraction and abdominal peristalsis (Sanders et al., 2012; Singh et al., 2014), and therefore could have antidiarrheal effects (Tradtrantip et al., 2010; Namkung et al., 2011b; Jiang et al., 2016). Finally, inhibition of renal TMEM16A could potentially lead to proteinuria and acidosis (Faria et al., 2014; Schenk et al., 2018). For activators of TMEM16A, opposite effects are possible, which is why local application via aerosol may be recommended for the treatment of CF lung disease. Nevertheless, oral application of a TMEM16A-blocker was shown to increase survival of CFTR-knockout mice (Walker et al., 2006).

## Activating or Inhibiting TMEM16A?

**Figure 7:**
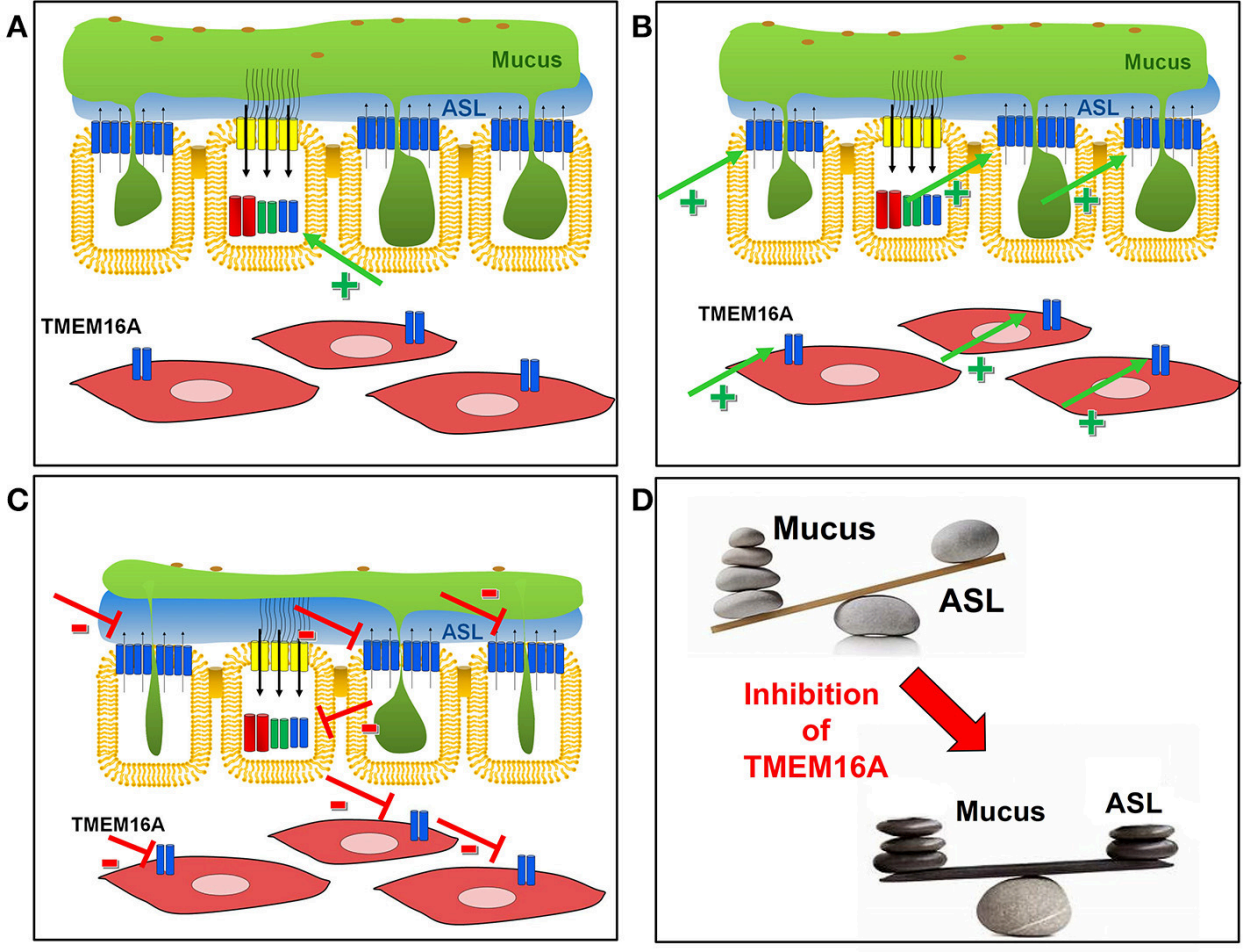
Activation of inhibition of TMEM16A in airways of CF patients. **(A)** Activators of TMEM16A (green arrow, +) will find only few TMEM16A channels in the apical membrane of ciliated epithelial cells, which could enhance fluid secretion by ciliated epithelial cells. **(B)** In contrast, activators of TMEM16A will prominently activate TMEM16A strongly upregulated in mucus producing cells and airway smooth muscle cells during airway inflammation. This leads to hypersecretion of mucus along with airway contraction. **(C)** Inhibitors of TMEM16A will have an only limited (adverse) effect on TMEM16A channels expressed (fluid secreting) ciliated epithelial cells. In contrast, inhibition of TMEM16 channels in mucus producing cells and airway smooth muscle cells will inhibit mucus production/secretion and induce bronchodilation. **(D)** Inhibition of TMEM16 channels are likely to restore the balance between mucus and fluid in CF airways.

Under physiological conditions, airway mucus represents an innate defense mechanism against pathogens. It traps inhaled pathogens and particles and is part of the mucociliary clearance (Knowles and Boucher, 2002). However, mucus becomes a serious problem when hypersecreted in inflammatory lung diseases, such as asthma, COPD, and CF (Dunican et al., 2018). Despite many pathological findings and pathogenic mechanisms proposed for CF lung disease (c.f. above), the single most prominent finding is the excessive overproduction of highly viscous mucus with adhesive and cohesive properties in CF patients (Button et al., 2018). It causes airway obstruction and a reduced mucociliary clearance, and it thereby drives chronic inflammatory lung disease (Fahy and Dickey, 2010). Thus, inhibition of mucus production/secretion is likely to be the most effective treatment of CF lung disease, normalizing the imbalance between excessive mucus secretion and reduced ASL (Figure 7). Our recent data show that TMEM16A and other TMEM16 proteins are essential for mucus production and basal secretion of mucus in airways and intestine (Benedetto et al., 2019). These findings open up a new avenue for the therapy of inflammatory airway diseases, and particularly for the treatment of airway mucus plugging and CF lung disease.

In contrast, activation of TMEM16A in CF to facilitate fluid secretion may be rather ineffective due to the reasons outlined above (Figure 7A). An earlier clinical trial was performed using stabilized dinucleotides (Denufosol) to induce purinergic Ca^2+^ dependent Cl^−^ secretion and to restore ASL with the goal of improving lung function in CF. However, the clinical trial failed to demonstrate any benefit of denufosol (Ratjen et al., 2012; Moss, 2013). As an adverse effect, the aerosol induced cough in 52% of all patients. It may be speculated that denufosol may have induced additional mucus secretion and even additional airway obstruction. Being strongly upregulated in inflammatory airway disease in secretory cells and in ASM, activation of TMEM16A could induce adverse effects by augmenting mucus secretion and bronchoconstriction (Figure 7B).

Inhibition of TMEM16A in CF may appear counterintuitive in the first place (assuming a potential inhibition of fluid secretion), however, the available data suggest otherwise. As outlined above, in CF TMEM16A membrane expression is compromised (Ruffin et al., 2013; Benedetto et al., 2017), Cl^−^ secretion through CFTR is missing (Namkung et al., 2010a; Billet and Hanrahan, 2013; Benedetto et al., 2017; Lerias et al., 2018), and most TMEM16A is expressed in mucus secreting cells (Benedetto et al., 2019). Therefore, inhibition of TMEM16A may not substantially lower fluid secretion. In contrast, interfering with mucus and cytokine secretion by blocking upregulated TMEM16A is likely to improve mucociliary clearance and to improve lung function (Figure 7C). Along this line, talniflumate, the anti-inflammatory pro-drug of the common TMEM16A-inhibitor NFA that was originally developed by Argentinian Laboratorios Bago, increased survival of CF mice remarkably (Walker et al., 2006). Talniflumate had been further developed by the former company Genaera, as a mucoregulator for cystic fibrosis, chronic obstructive pulmonary disease, and asthma. Sadly, phase II trials have never been finished due to the shutdown of the company (Knight, 2004). In a large number of studies, mucus production, and secretion, as well as airway constriction were inhibited by niflumic acid and other inhibitors of TMEM16A (Kondo et al., 2012; Yim et al., 2013; Lin et al., 2015; Danielsson et al., 2017; Miner et al., 2019). The inhibitors niflumic acid, CaCCinhAO1, T16Ainh-A01, 17 benzbromarone, or niclosamide have been examined *in vivo* as well as *in vitro* in the low micromolecular range. Despite voltage dependence of TMEM16A-inhibition by NFA and other inhibitors, *in vivo* application to airway epithelial cells maintaining their intrinsic hyperpolarized membrane voltage demonstrated remarkable biological effects. Similar has been observed when TMEM16A inhibitors were applied to tracheal ring preparations *ex vivo*. Presumably TMEM16A is partially active in the airways, as low levels of ATP in the airway surface liquid maintain a basal activity of TMEM16A.

Because TMEM16A currents are blocked by niflumic acid, the published reports suggest that TMEM16A is in charge of both production and secretion of mucus. Niflumic acid, however, is a rather non-specific drug that inhibits a number of ion channels and blocks intracellular Ca^2+^ signals (Cabrita et al., 2017). Suppression of Ca^2+^ signals by niflumic acid is probably due to inhibition of TMEM16A. Other TMEM16A inhibitors such as CaCCinhAO1, T16Ainh-A01, benzbromarone, or niclosamide also inhibit intracellular Ca^2+^ and mucus release (de La Fuente et al., 2007; Kondo et al., 2017; Miner et al., 2019). It is important to note that other TMEM16 paralogues are also blocked by inhibitors of TMEM16A (Sirianant et al., 2015; Wanitchakool et al., 2016). Because several TMEM16 paralogues are expressed in airway epithelial cells, the possible contribution of other TMEM16 proteins to Ca^2+^ signaling and mucus production/secretion is currently unknown. Using different TMEM16 knockout mice and TMEM16 knockout cell lines, we found that most TMEM16 paralogues affect intracellular Ca^2+^ signals (Kunzelmann et al., 2016; Cabrita et al., 2017). It is currently assumed that at least two TMEM16 members, TMEM16A and TMEM16F control mucus production and mucus secretion (Benedetto et al., 2019).

## Conclusion

Despite significant progress in the development of CFTR–specific treatments for CF lung disease, it appears reasonable to search for alternative drug targets in CF. Potentiators and correctors of mutant CFTR show benefit in patients carrying the common F508del mutation. Improvement of lung function by the recent combinatorial drugs can be as high as 13%. Insight into mode of action of these compounds is still limited, and the costs for treatment may exclude some patients from therapy (Ferkol and Quinton, 2015). Moreover, a fraction of patients with particular CFTR mutations [type 1,5,6,7 mutations (De Boeck and Amaral, 2016)] will not respond to such a treatment.

Restoration of the mucus/liquid balance has been the driving force behind the search for novel openers of secretory Cl^−^ channels (SLC26A9 and TMEM16A) and basolateral pro-secretory K^+^ channels, as well as for inhibitors of reabsorptive Na^+^ channels. The counterintuitive idea of using inhibitors of TMEM16 channels is based on their role for mucus production and mucus secretion, which were uncovered only recently. Overwhelming mucus production and mucus plugging is the central problem in CF lung disease. Therefore, potent and well-tolerated TMEM16-inhibitors, which have FDA-approval for other diseases, should be further examined in preclinical and clinical studies to be use in CF lung disease and other inflammatory airway diseases.

## Ethics Statement

All animals studies were approved by the local ethical board.

## Author Contributions

KK wrote manuscript, analyzed data, and designed experiments. RS, JO, and IC analyzed data, designed experiments, and performed experiments. TD, AB, and MJ wrote manuscript. RB wrote manuscript, analyzed data, designed experiments, and performed experiments.

### Conflict of Interest Statement

The authors declare that the research was conducted in the absence of any commercial or financial relationships that could be construed as a potential conflict of interest.
